# Comparison of Safety and Efficacy after Emergency Stenting in Patients Exhibiting Intracranial Atherosclerotic Stenosis Associated with Large-vessel Occlusion with and without Intravenous Infusion of Tirofiban

**DOI:** 10.1007/s00270-023-03372-7

**Published:** 2023-02-16

**Authors:** Rana Garayzade, Ansgar Berlis, Stefan Schiele, Hauke Schneider, Michael Ertl, Gernot Müller, Christoph J. Maurer

**Affiliations:** 1 Department of Diagnostic and Interventional Radiology and Neuroradiology, Augsburg University Hospital, Augsburg, Germany; 2 Institute of Mathematics, Augsburg University, Augsburg, Germany; 3 Department of Neurology, Augsburg University Hospital, Augsburg, Germany

**Keywords:** Tirofiban, Acute ischemic stroke, Endovascular treatment, Large artery atherosclerosis, Intracerebral hemorrhage, Rescue stenting, Bailout technique, Mechanical thrombectomy

## Abstract

**Purpose:**

Intracranial rescue stent angioplasty is a bailout strategy for acute stroke patients in cases of unsuccessful endovascular thrombectomy due to underlying atherosclerotic stenosis. However, there is no consensus on a preprocedural and intraprocedural antiplatelet regimen. The aim of this single-centre study was to compare the safety and efficacy of emergency stenting in patients exhibiting intracranial atherosclerotic stenosis-related acute large-vessel occlusion with or without peri-interventional intravenous infusion of tirofiban.

**Materials and Methods:**

We performed a retrospective analysis of 78 patients who were treated with rescuestent angioplasty between 2010 and 2019 due to acute ischaemic stroke. The patients were divided into 2 groups: those who received peri-interventional intravenous tirofiban and those who did not receive tirofiban. We compared clinical safety and functional outcomes in both treatment groups with symptomatic haemorrhage as the primary endpoint. Bivariate and multivariable logistic regression was performed to investigate the association between tirofiban and outcome measures.

**Results:**

Thirty-seven patients were treated with intravenous tirofiban (47.4%), and 41 patients did not receive intravenous tirofiban (52.6%). Statistical analysis revealed no significant difference between the two groups in the rate of symptomatic haemorrhage (16.2% in the tirofiban group versus 14.6% in the control group, *p* = 0.847). The 3-month mortality (21.6% in the tirofiban group versus 17.1% in the control group, *p* = 0.611) and good functional outcomes according to the modified Rankin scale (45.9% versus 34.1%, *p* = 0.289) were comparable.

**Conclusion:**

The results of our study suggest that the application of tirofiban for rescue stenting after failed mechanical thrombectomy is safe.

## Introduction

Several randomized clinical trials have shown the efficacy of endovascular thrombectomy over standard medical care in patients with acute ischaemic stroke caused by the occlusion of large vessels of the anterior circulation [1, 2]. However, acute stroke caused by in situ thrombosis at the site of intracranial atherosclerotic stenosis differs from stroke caused by embolic occlusion and may not respond as well to modern mechanical thrombectomy (MT) procedures [3].

Despite initially successful recanalization, patients with underlying atherosclerosis may develop immediate reocclusion of the target vessel by up to 40%, which is associated with poorer clinical outcomes [4–7]. In these cases, rescue stent angioplasty might be a treatment option to achieve permanent recanalization [8–13]. Retrospective data suggest that acute intracranial stenting is a safe and effective rescue strategy in patients with large-vessel occlusion (LVO) who fail mechanical thrombectomy (MT), potentially leading to better functional outcomes and lower mortality rates than those in patients with failed MT without rescue stenting [14].

However, there is no consensus on the periprocedural use of prophylactic antiplatelet agents during stent angioplasty in acute stroke, and the use of antiplatelet agents varies considerably [15–17]. The classical oral antiplatelet agents cannot be reversed easily and have a half-life of several days. The administration of heparin as an alternative seems to increase the risk of bleeding during endovascular stroke treatment [18].

Tirofiban is a highly selective, fast-acting glycoprotein IIb/IIIa (Gp IIb/IIIa) platelet receptor antagonist with a short half-life that can be given intravenously. Its administration in the systemic treatment of acute ischaemic stroke appears to be safe [15, 19–21], but data in this regard are sparse and inhomogeneous. Therefore, the aim of our study was to report our experience with the use of tirofiban as first-line antiplatelet therapy after rescue stenting for LVO due to atherosclerotic stenosis.

## Material and Methods

### Patient Selection

We included all patients admitted to our institution between 2010 and 2019 with acute ischaemic stroke due to intracranial LVO who were treated with rescue stent angioplasty. Patients with extra/intracranial tandem lesions were excluded.

Patients were divided into two groups: those who received peri-interventional tirofiban and those who did not receive intravenous tirofiban (control group).

### Intravenous rtPA Therapy (IVT) and EVT Procedures

Patients presenting within 4.5 h after stroke onset who were eligible for IV thrombolysis received IV rtPA as bridging therapy and MT as soon as possible. Treatment decisions for patients presenting > 4.5 h after stroke onset were based on CT perfusion mismatch and/or Alberta Stroke Program Early CT Score on noncontrast CT.

All endovascular thrombectomy procedures were performed using general anaesthesia. Stent retrieval and aspiration methods during EVT were selected at the discretion of the treating interventionalist. In cases of failed EVT due to underlying arterial stenosis, rescue treatment with stent placement and balloon angioplasty was performed. Generally, self-expanding stents with prior PTA or—in some cases in vertebrobasilar occlusion—balloon-mounted stents were used. In cases of inadequate perfusion, additional PTA was performed after stent placement.

### Postprocedural Imaging

A flat-detector CT scan was performed routinely immediately after mechanical thrombectomy within the angiography suite, and a head CT was performed 24 (± 6) hours after thrombectomy. In cases of neurological deterioration, an earlier CT scan was performed.

### Clinical and Imaging Assessment

The clinical outcome was assessed using the modified Rankin scale (mRS) score at discharge. Good outcome was defined by a modified Rankin scale (mRS) score of 0–2. Poor outcome was defined by an mRS score of 3–6. The safety outcomes were serious haemorrhage, 90-day good functional outcome and 90-day mortality.

Serious haemorrhage included symptomatic intracranial haemorrhage and other haemorrhage (gastrointestinal, etc.) that required intervention, such as blood transfusion or surgery. Symptomatic intracranial haemorrhage was defined as parenchymal haemorrhage (PH 1 and PH 2) diagnosed in the clinical setting of a worsening of 4 or more points on the NIHHS score [22].

### Administration of Tirofiban

We used a high-dose bolus regimen adopted from cardiology [23, 24]. Tirofiban was administered intravenously at a dose of 25 mcg/kg within 3 min during stenting followed by 0.15 mcg/kg/min and continued for up to 24 h after the procedure, when no obvious ICH or subarachnoid haemorrhage (SAH) was found on CT scans performed in the angiography suite immediately after the procedure. After follow-up CT at 24 (± 6) hours, tirofiban was usually substituted for ticagrelor or clopidogrel. If an earlier follow-up CT showed significant ICH or severe systemic bleeding, tirofiban infusion was terminated.

### Antiplatelet Treatment Regimens in the Control Group

In the control group, periprocedural antiplatelet treatment regimens were rather inhomogeneous before institutional standardization with tirofiban. Regimens consisted of ASA monotherapy (500 mg i.v.) in 7 cases (17,1%); ASA in different combinations with clopidogrel, heparin, ticagrelor or intraarterial (i.a.) rt-PA in 19 cases (46.3%); heparin monotherapy in 9 cases (22.0%); and clopidogrel alone or in combination with heparin and/or i.a. rt-PA in 3 cases (7.3%). Three patients (7.3%) received no antiplatelet medication. After endovascular therapy and intracranial stenting, dual antiplatelet therapy with a loading dose of clopidogrel/ticagrelor/prasugrel was started in 27 patients (65.9%); 4 patients received only aspirin, and 3 patients received only anticoagulant therapy.

### Statistical Analysis

Descriptive statistics were used to summarize patient characteristics and outcomes. Continuous variables are described as the median and interquartile range. Categorical variables are expressed as numbers and percentages. Variables were compared between the group with tirofiban and the group without tirofiban. We used a Wilcoxon rank-sum test for continuous variables and a Chi-squared test or Fisher’s exact test for categorical variables.

To investigate the association between tirofiban and different outcomes, we performed bivariate and multivariable logistic regression analyses for each outcome. The unadjusted model included only the use of tirofiban. The multivariable logistic regression was adjusted for age, sex and target artery occlusion.

All statistical analyses were performed with R 4.1.1, and a *p* value < 0.05 was considered statistically significant.

## Results

Seventy-eight of 1154 endovascular-treated stroke patients met the inclusion criteria. Twenty-one patients (51.2%) were treated with i.v. rt-PA prior to EVT in the control group, and 11 patients (29.7%) were treated thusly in the tirofiban group. Thirty-seven patients received peri-interventionally intravenous tirofiban (47.4%), and 41 patients did not (52.6%). The baseline characteristics of the patients are shown in Table 1.

**Table 1:** Baseline characteristics of the study population

	No tirofiban (*n* = 41)	Tirofiban (*n* = 37)	*p* value
Age (median) (IQR) (yr)	73.3 (64.2–78.2)	70.6 (63.7–74.7)	0.467
Sex (male)	21(51.2%)	27(73.0%)	0.049
mRS at admission (median) (IQR)	4.0 (4.0–5.0)	5.0 (4.0–5.0)	0.782
Prior antiplatelet therapy	14 (34.1%)	11 (29.7%)	0.676
Prior anticoagulation	5 (12.2%)	1 (2.7%)	0.204
i.v. rtPA before EVT	21(51.2%)	11 (29.7%)	0.054
Target artery occlusion			0.148
Terminal ICA	10 (24.4%)	3 (8.1%)	
MCA M1	17 (41.5%)	20 (54.1%)	
Vertebral V4 Segment and basilar	14 (34.1%)	14 (37.8%)	

The median age was 70.6 (IQR 63.7–74.7) in the tirofiban group and 73.3 (IQR 64.2–78.2) in the control group. There was no statistically significant difference in terms of age, mRS score on admission or target artery occlusion between the two groups. There was, however, a borderline significant sex difference between the two groups, with 73.0% male patients in the tirofiban group and 51.2% male patients in the control group (*P* = 0.049).

Additional peri-interventional antiplatelet or heparin therapy in the tirofiban group was selected by the treating interventionalist depending on prior antiplatelet or anticoagulation medication, as shown in Table 2.

**Table 2:** Prior antiplatelet or anticoagulation therapy in the tirofiban group

	Heparin plus Tirofiban	ASA (100 mg) plus Tirofiban	ASA (500 mg) plus Tirofiban	ASA plus Heparin plus Tirofiban	Tirofiban only	ASA plus i.a. rt-PA plus Tirofiban
With antiplatelet or anticoagulation premedication	1 (ASA)	0	1 (Apixaban)	0	10 (2 cases with dual antiplatelet therapy in premedication and 8 cases with premedication with ASA alone)	0
No premedication	1	8	10	2	2	2

### Primary Endpoint: Serious Haemorrhage

In the tirofiban group, 6 patients (16,2%) suffered from serious bleeding complications. Symptomatic intracranial haemorrhage was observed in 4 patients, gastrointestinal bleeding and consecutive endoscopic clipping in 1 patient and gastrointestinal bleeding with haemorrhagic shock and red blood cell transfusion in another patient. Infusion of tirofiban was terminated in two patients immediately after flat-detector CT within the angiography suite due to subarachnoid haemorrhage. One of these patients died, not because of haemorrhage but due to subsequent stent occlusion and consecutive extensive infarctions.

In the nontirofiban group, 6 patients (14,6%) suffered from serious haemorrhagic complications. Four patients had symptomatic intracranial haemorrhage, one patient had severe nosebleeds requiring tamponade and red blood cell transfusion, and one patient had an aneurysma spurium after femoral puncture requiring surgical therapy and red blood cell transfusion.

We observed serious thrombocytopenia (platelet count < 90,000 cells/µl) in 2 cases (5.4%) in the tirofiban group, however, without clinical sequelae.

Our analysis revealed no statistically significant difference between the two groups in the rate of serious haemorrhage (Table 3).

**Table 3:** Comparison of the outcomes in the tirofiban and control group

	No tirofiban *n* = 41 (52.6%)	Tirofiban *n* = 37 (47.4%)	Adjusted	*p*-value adjusted
3-month good outcome	14 (34.1%)	17 (45.9%)	1.84 [0.69–4.92]	0.224
3-month death	7 (17.1%)	8 (21.6%)	1.79 [0.51–6.26]	0.359
serious haemorrhage	6 (14.6%)	6 (16.2%)	0.96 [0.27–3.44]	0.949
any haemorrhage	7 (17.1%)	13 (35.1%)	2.36 [0.79–7.08]	0.126

Multivariable analysis revealed no association of tirofiban with serious haemorrhage (adjusted OR [aOR], 0.96; 95% CI, 0.27–3.44; *P* = 0.949).

Secondary endpoints: good functional outcome and mortality at 90 days.

The rate of good functional outcome at 3 months (mRS 0–2) was numerically higher in the tirofiban group than in the control group, 17 versus 14 patients, but the difference was not statistically significant (45.9% versus 34.1%, *P* = 0.224).

There was no statistically significant difference between the two groups in mortality (21.6% in the tirofiban group versus 17.1% in the control group, *p* = 0.611). Multivariable analysis revealed no association of tirofiban with good outcome (aOR, 1.84; 95% Cl, 0.69–4.92; *P* = 0.224) or death at 3 months (aOR, 1.79; 95% CI, 0.51–6.26; *P* = 0.359).

### Primary and Secondary Endpoints in Treatment Subgroups of the Control Group

We used ASA with clopidogrel, heparin, ticagrelor and i.a. rtPA in various combinations in 19 cases (47%) in our control group. Further analysis revealed a tendency towards a higher rate of serious haemorrhage in subgroups with a combination of multiple anticoagulant drugs (Table 4).

**Table 4:** Safety and clinical outcome in treatment subgroups of the control group (*n* = 41)

Drug combination in control group	Serious haemorrhage	Good outcome	Death
ASA alone in 7 cases (17%)	0 (0.0%)	0 (0.0%)	3 (42.9%)
ASA with clopidogrel, heparin, ticagrelor and i.a. rtPA in different combinations in 19 cases (47%) considering previous antiplatelet and anticoagulant therapy	4 (21.1%)	8 (42.1%)	3 (15.8%)
Heparin alone in 9 cases (22%), of which in 4 cases immediately after EVT loading dose with 600 mg clopidogrel via gastric tube	0 (0.0%)	5 (55.6%)	0 (0.0%)
Clopidogrel alone or in combination with heparin and/ or i.a. rtPA in 3 cases (7%)	1 (33.3%)	1 (33.3%)	0 (0.0%)
No medication in 3 cases (7%)	0 (0.0%)	0 (0.0%)	1 (33.3%)

### Stent Occlusion

Follow-up Doppler ultrasound data were available for 20 patients (54.1%) in the tirofiban group and for 19 patients (46.3%) in the nontirofiban group. Available ultrasound data showed that the stent was open in 18 cases (43.9%) in the nontirofiban group and in 19 cases (51.4%) in the tirofiban group. Stent occlusion was observed in 1 patient (2.4% versus 2.7%) in each group.

### Subgroup Analysis: Comparison of Tirofiban and ASA Monotherapy

We compared the group of patients receiving ASA alone with the group of patients receiving tirofiban alone. In the group receiving tirofiban alone, a good functional outcome was observed in 41.7% (5 of 12), whereas no good outcome was observed in the group receiving ASA alone. Tirofiban was associated with lower mortality (25% versus 42.9%). However, due to the small number of cases, no statistical analysis could be performed.

## Discussion

The rate of failure of MT in the treatment of acute ischaemic stroke ranges between 10 and 30% with a strong negative effect on the clinical outcome of these patients [25]. Intracranial stenosis related to atherosclerotic disease seems to be a major reason for failed MT, but some patients seem to benefit from intracranial rescue stenting in these cases [15, 19–21]. However, a major problem remains the adequate antiplatelet regimen for these patients. Medical therapy varies between interventionalists and institutions, and no standardization or guideline exists for neuroradiological procedures regarding antiplatelet therapy in patients exhibiting acute stroke [17].

Tirofiban can be administered intravenously and has a relatively short half-life. To date, the drug has only been approved for the treatment of unstable angina pectoris and non-Q-wave myocardial infarction. Due to its favourable properties, however, it is also increasingly used for neuroradiological interventions [26–28].

Our single-centre study of a small cohort of patients with intracranial rescue stenting after failed MT suggests that peri-interventional administration of tirofiban, mainly given in addition to preexisting antiplatelet therapy or in combination with other antiplatelet or anticoagulation medication, does not significantly increase the risk of severe haemorrhage in stroke patients with intracranial stenting.

These results are consistent with the primary safety outcome of the recent multicentre RESCUE BT trial among patients with acute ischaemic stroke due to large-vessel occlusion undergoing endovascular thrombectomy and treatment with intravenous tirofiban compared with placebo [29]. In this trial, no significant difference was reported in the incidence of symptomatic intracranial haemorrhage between the groups.

Several retrospective studies and case series have demonstrated a favourable effect of the systemic administration of tirofiban on patients with intracranial dissection [30] and on patients after emergency angioplasty with and without intracranial stenting [31, 32]. The main advantages of the drug are its ease of administration, rapid onset of action and short half-life. In addition, tirofiban infusion can be stopped in cases of haemorrhagic complications or before imminent surgery, and due to the shorter half-life, the platelet inhibition is reversed much faster than it would be after oral antiplatelet therapy. Platelet aggregation returns to near baseline levels within 4 to 8 h after cessation of a tirofiban infusion, a finding consistent with the drug’s elimination half-life of ≈2 h [33]. In contrast, ASA causes irreversible inhibition of platelet aggregation which persists for 5–7 days after aspirin discontinuation, which is why aspirin withdrawal is required 5 days before high-risk bleeding procedures to allow platelets to recover [34].

Platelets seem to play a major role in acute stent occlusion [35], so fast, specific and safe antiplatelet strategies are necessary in cases of acute LVO in intracranial atherosclerotic stenosis. Therefore, tirofiban seems to be the ideal agent for preventing thrombus formation, since the binding of platelet GpIIb/IIIa receptor to fibrinogen is the final step for platelet aggregation [36].

Intravenous ASA, on the other hand, is popular in neurointerventional practice. Inhibition of platelet function is evident within 1 h, although even with optimal ASA therapy, the platelet inactivation caused by ASA is only partial [37].

In our control group, ASA 500 mg i.v. was given as a single antiplatelet agent in 7 (17%) cases. We did not observe a good outcome in any of these patients, although none of these patients had a serious haemorrhage. Although we do not have data on stent patency for this subgroup, the poorer outcome and associated large infarcts might be caused by insufficient antiaggregation with consecutive stent occlusion. Overall, 19 patients received monotherapy, either with tirofiban (*n* = 12) or ASA (*n* = 7). The patients in the tirofiban group had no serious haemorrhage, whether or not i.v. rtPA was administered before EVT, and good outcomes were more common than they were seen in the ASA-only group. These results suggest that tirofiban as a sole antiplatelet agent appears to be less likely to cause major bleeding but may lead to a better outcome and a trend towards low mortality compared with the corresponding effects of ASA. However, a statistical statement is not possible due to the small sample size.

We used ASA with clopidogrel, heparin, ticagrelor and i.a. rtPA in various combinations in 19 cases (47%) in our control group. When ASA was combined with other medications, it seemed to increase the risk of serious haemorrhage, consistent with published literature [17].

Our study has several limitations. First, its overall sample size is small, which may have a considerable impact on the results. Second, this is a retrospective study with obvious constraints. In particular, the use of antiplatelet agents in the control group was rather inhomogeneous before institutional standardization with tirofiban as a first-choice antiplatelet, and sufficient follow-up was limited. However, our tirofiban protocol has been used consistently since its initiation and therefore provides a relatively homogenous dataset. Further research and standardization of practice across centres are essential to address the question of the ideal antiplatelet regimen.

## Conclusions

Our study suggests that the use of tirofiban in cases of rescue stenting after failed mechanical thrombectomy is safe and could lead to a good functional outcome.

## Abbreviations

mRs: Modified Rankin score
ASPECTS: Alberta Stroke Program Early CT Score
PTA: Percutaneous balloon angioplasty
CT: Computed tomography
NIHSS: National Institutes of Health Stroke Scale
