# Correction to: Comparison of Safety and Efficacy after Emergency Stenting in Patients Exhibiting Intracranial Atherosclerotic Stenosis Associated with Large-vessel Occlusion with and without Intravenous Infusion of Tirofiban

**DOI:** 10.1007/s00270-023-03413-1

**Published:** 2023-03-13

**Authors:** Rana Garayzade, Ansgar Berlis, Stefan Schiele, Hauke Schneider, Michael Ertl, Gernot Müller, Christoph J. Maurer

**Affiliations:** 1 Department of Diagnostic and Interventional Radiology and Neuroradiology, Augsburg University Hospital, Augsburg, Germany; 2 Institute of Mathematics, Augsburg University, Augsburg, Germany; 3 Department of Neurology, Augsburg University Hospital, Augsburg, Germany

**Correction to: Cardiovasc Intervent Radiol** https://doi.org/10.1007/s00270-023-03372-7

Unfortunately, Table number 3 in the original article was not formatted correctly during the publication process. Please see the correct version of Table 3 here. The original article has been corrected.

**Table 3:** Comparison of the outcomes in the tirofiban and control group

	No tirofiban *n* = 41 (52.6%)	Tirofiban *n* = 37 (47.4%)	Adjusted	*p* value adjusted
3-Month good outcome	14 (34.1%)	17 (45.9%)	1.84 [0.69–4.92]	0.224
3-Month death	7 (17.1%)	8 (21.6%)	1.79 [0.51–6.26]	0.359
Serious hemorrhage	6 (14.6%)	6 (16.2%)	0.96 [0.27–3.44]	0.949
Any hemorrhage	7 (17.1%)	13 (35.1%)	2.36 [0.79–7.08]	0.126

